# Photoaging: Reversal of the Oxidative Stress Through Dietary Changes and Plant-Based Products

**DOI:** 10.7759/cureus.37321

**Published:** 2023-04-09

**Authors:** Reet Hooda, Bhushan Madke, Ankita Choudhary

**Affiliations:** 1 Dermatology, Jawaharlal Nehru Medical College, Datta Meghe Institute of Medical Science, Wardha, IND; 2 Dermatology, Venereology, and Leprosy, Jawaharlal Nehru Medical College, Datta Meghe Institute of Medical Science, Wardha, IND; 3 Dermatology, B.J. Medical College, Ahmedabad, IND

**Keywords:** photoprotection, ultraviolet radiation, oxidative stress, ros, photoaging

## Abstract

Redox flagging represents all life processes, and maintaining a physiological level of antioxidants is essential for the legitimate working of the cell. Genetics and environmental triggers are two major culminating factors for skin aging, both chronological and photoaging. The latter, however, relies principally upon the level of ultraviolet radiation (UVR) exposure and the skin phototype. Apart from causing DNA damage, UVR also stimulates the receptors present in keratinocytes as well as fibroblasts. This in turn leads to the breakdown of collagen and a breach in the generation of new collagen. It is speculated that the breakdown of collagen in the dermis is ensured by the defective restoration that ultimately hampers the structural integrity of skin, leading to wrinkled and atrophic skin. The skin has an admixture of various endogenous antioxidants that work synergistically with vitamins and minerals to maintain cellular equilibrium. Although, their role in safeguarding the cells against the detrimental effects induced by UVR is still questionable and requires further research. However, the advancement in the biology of skin has led to the development of strategies that aim at skin rejuvenation and retarding the progression of photoaging and its visible signs. Photoaging in this article is reviewed in light of current concepts in pathogenesis and its prevention. In addition, the article focuses on both prevailing and forthcoming treatment strategies primarily through plant-based products that will help slow down the process of photoaging.

## Introduction and background

Photoaging is an amalgamation of various distinctive clinical, histopathological, and functional cutaneous changes occurring due to chronic sun exposure. Heliodermatosis, actinic dermatosis, and accelerated skin aging are a few synonyms commonly used for photoaging [1]. Photoaging depends on two major factors: (1) the amount of UV exposure and (2) the quantity of melanin in the skin. This is in contrast to chronological aging, which primarily depends on physiological predisposition. The spectrum of cosmic radiation reaching the earth includes ultraviolet radiation (UVR) with a wavelength ranging from 290 to 400 nm, visible light having a wavelength between 400 and 760 nm, and infrared radiation (IR) having wavelengths from 760 to 1 mm. The five mutually dependent mechanisms of photoaging include free radical generation, defective DNA repair, genomic instability, epigenetic dysregulation, and proteostasis imbalance.

## Review

**Figure 1:**
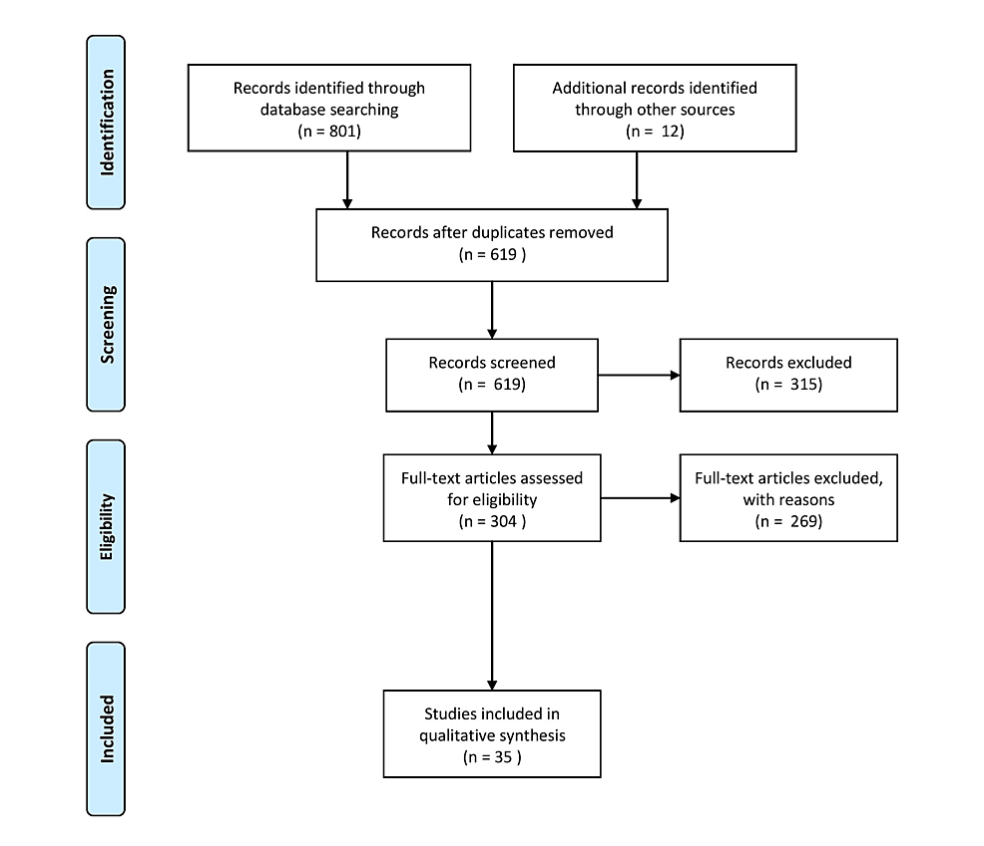
PRISMA flow diagram

A diligent search of the PubMed and Google Scholar databases was made. A PubMed search was done with the keywords "photoaging" in combination with "oxidative stress." Articles were included with the time duration limited to the last five years. All the titles and abstracts were screened for their relevance and duplication. The PRISMA flow diagram of included studies is shown in Figure 1.

Effects of UVR on cells and tissues

UVR is a major contributor to photoaging, of which UVC radiation, due to its near complete absorption by the ozone layer, hardly causes any detrimental effect on the skin. The other two spectrums of UVR, i.e., UVA (320-400 nm) and UVB (290-320 nm) have a role in the etiopathogenesis of photoaging. Since UVA has a higher wavelength, it penetrates deeper, directly affecting both dermal fibroblasts and epidermal cells. UVB has a shorter wavelength than UVA and acts preferentially over the epidermal components where it affects the melanocyte and keratinocyte DNA; however, indirect consequences have also been seen on the dermis due to the production of proteolytic enzymes. Due to its lesser penetration, UVB causes acute skin reactions in the form of sunburn. High UVA doses may be received indoors, while erythemal UVB radiation is altered [2]. UVB instigates damage to the DNA directly via interplay with epidermal cytokines, which results in mutagenesis and the production of cyclobutane dimers and pyrimidine-pyrimidone photoproducts [3]. UVA leads to the indirect generation of reactive oxygen species (ROS), which ultimately culminates in the breakage of DNA strands. ROS produce hydrogen peroxide, superoxide anion, singlet oxygen, hydroxyl radicals, and nitric oxide [4].

The lipid peroxidation caused by free radicals gives rise to prostaglandins via phospholipase A [5]. The generation of ROS, in turn, causes the activation of nuclear factor kappa-B (NF-kB) and the mitogen-activated protein kinase (MAPK) pathway. This leads to the up-regulation of pro-inflammatory cytokines and various growth factors such as interleukin-1, tumor necrosis factor, and epidermal growth factor. The transcription factor precursors c-Fos and c-Jun heterodimerize to form the transcription factor activator protein 1. All of these heighten the activity of the matrix metalloproteinases (MMPs), leading to dermal extracellular matrix degradation and a reduction in collagen synthesis [6]. The visible signs related to an intrinsic decrease in collagen are fine lines, xerosis, and lax skin [7]. Since UVR aggravates the shortening of telomeres and hence favors the concept that chronological aging is often superimposed upon photoaging, DNA damage in the skin cells undergoes repair by two different mechanisms: nucleotide excision repair (NER) and base excision repair (BER) [8]. The DNA damage induced by ROS undergoes repair primarily by the BER system, while the direct damage caused by the influence of UVR on DNA occurs mainly by the NER system [9].

Effects of visible light and infrared radiation

Recent emerging studies have shown visible light (400-770 nm wavelength) and IR (770-1 mm) to also be implicated in the causation of photoaging. IR diminishes the production of collagen type 1 via its effect on procollagen 1 stimulating TGF-β in the human skin [10]. IR causes an increase in the number of cutaneous mast cells, and this is an essential histological feature of photoaging [11]. IR leads to the stimulation of MMPs via the production of ROS, which in turn causes collagen degradation [12]. Exposure to visible light has been shown to induce erythema and pigment darkening within 24 hours. This induced pigmentation was darker and more sustained than that caused by UVA1. Melanin responsible for skin darkening has two major types: eumelanin and cysteine-rich pheomelanin, found in lighter phenotypes. Eumelanin is the one that offers greater photoprotection as compared to pheomelanin [13]. In fact, pheomelanin on UVR exposure can act as a photosensitizer by inciting lipid peroxidation and ROS production [14]. Hence for melanoma, pheomelanin is considered to have a weaker carcinogenic potential.

Melanin serves as a protective factor by absorbing the harmful UVR and protecting the skin from its deleterious effects. This is the reason why dark-skinned individuals encounter photoaging later than people with lighter skin [15]. Hence induction of pigmentation in the skin by thymine dinucleotide sequence motifs can be one novel strategy for delaying the progression of photoaging [16]. Tanning is also one of the ways the skin tries to safeguard its internal biochemical reactions from UVR [17]. Few organic and inorganic compounds, owing to their particulate nature on the nanoscale, closely mimic melanin in terms of absorbing and scattering UVR. Oxybenzone, octinoxate, avobenzone, octyl salicylate, para-aminobenzoic acids, and titanium dioxide are essential ingredients in sunscreen [18]. UVR causes stimulation predominantly via adenylate cyclase pathways. There is the synthesis of the alpha-melanocyte-stimulating hormone, which in turn binds to the melanocortin 1 receptor (MC1R) on the melanocyte surface, eventually leading to pigmentation. Antioxidants cause potentiation of the MC1R pathway, leading to two significant effects: (1) reduction in the hydrogen peroxide levels and (2) stimulation of the NER pathway on exposure to UVR [19].

Role of antioxidants

Antioxidants can be both endogenous and exogenous (obtained from the diet) and can be either natural or synthetic. The skin has a broad spectrum of endogenous antioxidants, both enzymatic and non-enzymatic, like vitamin E; vitamin C; bilirubin; uric acid; ubiquinol; iron; and copper binding extracellular proteins such as haptoglobin, albumin, and transferrin [20]. During normal conditions, the epidermis has high enzymatic antioxidant expression, but the dermis has a low baseline level. Contrary to photoaged skin, these antioxidants are found endogenously and are significantly lacking in the stratum corneum. The upper dermis showed a collection of modified oxidized proteins. Antioxidants can act as the first and second lines of defense with different mechanisms. In the former, it suppresses the generation of ROS, while in the latter, it affects the chain initiation and propagation reactions of ROS free radicals [21].

Natural ingredients as part of treatment for photoaging

Nutrition is an important factor that aids the skin's antioxidant defense status. Natural health products are used to treat photodamage functions by stimulating new cell formation or impeding the biochemical processes that hamper the cutaneous architecture. Topical application of botanical extracts like green and white tea extracts can have immunomodulatory effects on the damage caused by UVR [22]. Judicious intake of vitamins, polyunsaturated fatty acids, and polyphenols can prevent various age-related diseases. Prior skin treatment with antioxidant lotions can be an essential way of preventing age-related oxidative damage [23]. There are three primary sources of natural antioxidants: polyphenolic compounds, carotenoids, and vitamins. The main sources of polyphenols with antioxidant activity are shown in Table 1.

**Table 1:** Main sources of polyphenols with antioxidant activity The author has recreated the table from the source [24].

Polyphenol	Major sources	Mechanism of action
Flavonoid		
Catechins	Tea	Decreases UV-induced changes in the epidermis
Isoflavones: genistein, silymarin	Soy, thistle	Photoprotective ROS reduction
Proanthocyanidins (tannins)	Grape seed	Prevents tumor induction in response to UVR anti-inflammatory properties
Anthocyanins	Pomegranate	Antioxidant
Non-flavonoid		
Phenolic acids		
Benzoic acids: gallic acid, cinnamic acids	Grape and derivatives, tea, polypodium leucotomos	Antioxidant, anti-inflammatory, immunomodulating activities
Stilbene	Grape and derivatives	anti-aging, anti-photocarcinogenic properties
Resveratrol	Nuts, peanuts, grape seeds	Modulate cytokines and stimulate the expression of heat shock proteins, delays skin aging by blocking apoptotic events and mitochondrial dysfunctions

Eucomic and piscidic acids are the two crucial phenolic compounds responsible for the protection of keratinocytes against UVA-induced oxidative stress and apoptosis [25]. Caffeic and ferulic acids are the two critical antioxidant phenolic acids found in Polypodium leucotomos extracts obtained from tropical fern, which exhibit antioxidant, anti-inflammatory, and immunomodulatory properties [26]. The various action mechanisms include preventing lipid peroxidation and UVR-induced membrane damage, preventing keratinocyte apoptosis, inhibiting UV-mediated actin disarray, modulation of MMP activity by inducing tissue inhibitors of metalloproteinase, and prevention of transcriptional activation of proinflammatory cytokines and NF-κB factors.

The non-phenolic phytochemicals with photoprotective activity include carotenoids, caffeine, and sulforaphane (SFN). Carotenoids are pigments that plants generate. Β-carotene, lycopene, canthaxanthin, and lutein are abundant carotenoids in tomatoes, carrots, and algae. Carotenoids impede the activation of caspases and decrease the formation of breaks in the DNA strands following exposure to UVR, scavenging ROS. Lycopene has been shown to protect humans against UVR-induced erythema, and it is the most vital reducing agent. It can reduce the cations of lutein and zeaxanthin but cannot mitigate β-carotene [27]. Caffeine, found in huge amounts in coffee, has been found to alleviate the incidence of carcinoma of the skin by increasing the apoptosis of cells with defective DNA repair [28]. SFN is predominantly found in broccoli. The extracts of SFN get metabolized into isothiocyanates, which have decreased UVR-induced erythema in human skin [29].

Vitamins and diet

Vitamin C, also known as ascorbic acid, is an exogenous antioxidant since it cannot be generated in the human body and must be taken in the diet. Acerola, orange, lemon, tangerine, and tomato are a few fruits that are rich sources of ascorbic acid [30]. It acts as a photoprotectant by mitigating the ongoing inflammation in the skin; it also prevents peroxidation of lipids and protects the keratinocytes from undergoing apoptosis. The topical application of mixtures containing vitamin C has been shown to downregulate the levels of MMP, improving the clinical appearance of photoaged skin and having an anti-pigmentary effect. The stability of vitamin C is a prime area of concern and is widely influenced by temperature and concentration. Vitamin E is a lipid-soluble vitamin found predominantly in legumes and cereal grains. It has four significant isomers, of which the body can absorb only alpha-tocopherol. It exerts anti-inflammatory action due to its close collaboration with the eicosanoid system. The richest sources are spinach, whole grains, olive oil, nuts, and sunflower oil. Alpha-tocopherol is the most abundant vitamin E derivative in human tissues and sera [31]. Vitamin E has been shown to stabilize the skin barrier.

Synthetic antioxidants

There are two major classes of synthetic antioxidants: (1) nitroxides, which are mimetics of superoxide dismutase, and (2) coenzyme Q analogs. Tempol is the bioactive compound of nitroxides, and it causes protection from UVR-induced damage, prohibits the degradation of the extracellular matrix, and preserves the mechanism of collagen synthesis. On the other hand, idebenone serves as a bioactive compound of coenzyme Q, and it plays a role in safeguarding the cell from ROS by decreasing oxidative stress; it also diminishes the formation of sunburn cells. Coenzyme Q when applied topically has been shown to prevent photoaging by increasing the production of basement membrane components in the skin and reducing the oxidation level measured by weak photon emission [32].

Photoprotection and use of sunscreens

Photoprotective measures, like the use of sunglasses, garments to cover up, and hats, take a lead among all different actions regarding heliodermatosis. Since the upcoming studies have shown the implication of visible and infrared rays in causing photoaging apart from the UVR, consistent proper use of sunscreen needs to be emphasized now. A broad-spectrum sunscreen (90% UV absorbance occurs at ≥370 nm) having SPF30 offers a considerable amount of protection against UVR and visible light, thus helping to retard the progression of photoaging [33]. Currently, titanium dioxide and zinc oxide are the only two FDA-approved inorganic UV filters in category I - “GRASE” (generally recognized as safe and effective). The organic UV filters like cinnamates, benzophenones, salicylates, and PABA derivatives are either category II non-GRASE or category III and require further evaluation [34]. Sunscreen, if recommended, can reduce 55% of the ROS formation; however, adding antioxidants to sunscreen increases it beyond 55% [35].

## Conclusions

Skin being a complex, dynamic organ is constantly under the pelting of various environmental triggers, including UVR. In order to withstand the harsh insults, various protective factors like stratum corneum, melanin, and anti-inflammatory immunological responses do exist. But with time the damage caused by UVR can surpass the different protective mechanisms, which can lead to photoaging initiation. Generation of oxidative stress, depletion of natural antioxidants, shortening of telomeres, mitochondrial mutations, increased apoptosis of keratinocytes and fibroblasts, and activation of MMP are a few cornerstone factors in the causation of photoaging. Detailed knowledge regarding the pathomechanics of photoaging provides insight into the various strategies for photoprotection, and photoprotection is the primary treatment for photoaging. The use of various exogenous antioxidants and strengthening cellular antioxidant balance through proper nutrition are a few secondary treatment options that can reduce the incidence of photoaging.
